# Males differ more between developmental stages than females, and plasticity to light is stage-dependent in the tropical plant *Marchantia inflexa*

**DOI:** 10.1093/aobpla/plaf010

**Published:** 2025-02-22

**Authors:** Jonathan D Moore, D Nicholas McLetchie

**Affiliations:** Division of Natural Science, Campbellsville University, 1 University Drive, Campbellsville, KY 42718, United States; Department of Biology, University of Kentucky, 101 T.H. Morgan Building, Lexington, KY 40506-0225, United States

**Keywords:** *Marchantia inflexa*, sexual dimorphism, developmental stage, plasticity, pigments, chlorophyll, carotenoids, dry matter content, specific leaf area

## Abstract

Plants have evolved strategies to maintain photosynthesis and mitigate tissue-damaging high light. In some dioecious seed plants, these strategies are sexually dimorphic and are linked to spatial segregation of the sexes (SSS) along light gradients. In vascular tissue-free plants (bryophytes) with separate sexes, SSS is common, but how light gradients, sexual dimorphisms, and SSS correlate is not well understood. To test if sexual dimorphisms in vegetative or sexual stages lead to light-associated SSS in bryophytes, we used *Marchantia inflexa* whose males occupy a wider range of light conditions, including higher light conditions, than females. We also tested if changes in development differed between sexes. We grew 25 males and 25 females in a glasshouse with clones in low and high light and assessed pigment and biomass allocation traits in vegetative and sexual thalli (analogous to leaves), representing non-sexual and sexually reproductive stages. We expected males to exhibit traits consistent with high light acclimation more than females and greater sex differences in sexual thalli due to specialization. Further, we reasoned that males would change more between stages than females. For sexual thalli, males had higher carotenoid/chlorophyll ratios (consistent with expectation), while females had higher chlorophyll *a/b* ratios and dry matter content (opposite from expectations). Vegetative thalli were not sexually dimorphic but were more plastic to light than sexual thalli. Overall, the stages differed more for males than females, but without regard for light. However, female stages differed more for dry matter content. Males generally need greater change in pigmentation and biomass allocation than females between stages, and we posit links for individual traits to sex function. Specialization in sexual thalli constrains their plasticity to light compared to vegetative thalli. Yet, neither sexual dimorphism in sexual thalli nor greater change between stages for males than females clearly leads to light-associated SSS.

## Teaser

Assessing how sex, developmental stage, and light affected pigmentation and biomass allocation in *Marchantia inflexa* revealed that males generally differ more between stages than females, and that pigmentation and biomass allocation responses to light were stronger in vegetative than sexual stages. The former comparison increases the likelihood of identifying sex-specific patterns even when traits do not appear sexually dimorphic within a given stage.

## Introduction

While light is critical for photosynthetic organisms, excessive amounts damage living tissue. Thus, these organisms have evolved strategies to maintain adequate photosynthesis in lower light and also mitigate high-light-associated stresses, and these strategies often affect their distribution along light intensity gradients (Osmund *et al.* 1987; Gauslaa and Solhaug 1996; Dangremond *et al.* 2015). For non-mobile organisms, such as plants (Huey *et al.* 2002) and lichens (e.g. Tretiach and Brown 1995; Mafole *et al.* 2017), anatomical, morphological, and physiological traits are key in adjusting to their light environment. Furthermore, for dioecious seed plants, sex-specific traits as a consequence of sex function can lead to spatial segregation of the sexes (SSS) along light intensity gradients (Wade *et al.* 1981; Bierzychudek and Eckhart 1988; Dawson and Geber 1999; Nuñez *et al.* 2008; Vandepitte *et al.* 2009; Ortiz-Pulido and Pavón 2010; Matsushita *et al.* 2016). However, though SSS often occurs in vascular tissue-free plants (bryophytes) with separate sexes (e.g. Cameron and Wyatt 1990; Bowker *et al*. 2000), the interactive effects of light intensity gradients and plant traits on SSS are not as well characterized as in seed plants (but see Fuselier and McLetchie 2004; Castetter *et al.* 2019).

Established patterns in vascular plants provide good target traits (some by analogy) for investigating sex-specific light gradient interactions in bryophytes. These include leaf traits associated with species-level distributions along light gradients and some sex-specific patterns. In general, vascular plants that are adapted or can acclimate to high light intensities exhibit a suite of characteristics that can be grouped in two categories: pigmentation traits related to light capture/protection and carbon allocation traits related to photosynthetic organ morphology and biomass distribution. In particular, high light traits include decreases in photosynthetic pigments per unit mass (or shifts in their ratios), increased photoprotective pigment concentration, greater leaf thickness (lower specific leaf area [SLA], i.e. less photosynthetic area can support more biomass), increased leaf dry matter content (ratio of dry to fresh mass; Poorter *et al.* 2019), and changes in leaf shape (Givnish 1988; Sarijeva *et al.* 2007; Niinemets 2010). Due to sex-specific selection pressures, males and females sometimes differ in these traits (e.g. leaf size/shape/anatomy: Li *et al.* 2007; Korgiopoulou *et al.* 2019; Midgley 2010; pigmentation: Said *et al.* 2013; Rabska *et al.* 2020; Muñoz *et al.* 2021), which may lead to sex-specific interactions with environmental gradients, including light (Dawson and Geber 1999; Zhou *et al.* 2019; Zhang *et al.* 2020). Furthermore, sex-specific photosynthetic traits (e.g. higher female photosynthetic rate) are often linked to differences in sexual reproductive costs, which are higher for females after seed and cone/fruit investment (Obeso 2002). However, sexually dimorphic traits can be present before sexual reproductive investment (Quinn and Meiners 2008; Vega-Frutis and Guevara 2009).

Several trait patterns in bryophytes, which we discuss below, mirror those in seed plants and also provide targets for investigations of sex-specific light gradient relationships. In several bryophytes, high-light-associated traits include reduced chlorophyll, increased UVB-protective pigments, and increased carotenoids such as xanthophylls relative to chlorophyll (Hamerlynck *et al.* 2002; Dunn and Robinson 2006; Tobias and Niinemets 2010; Glime 2017; Fan *et al.* 2020). In the thalloid liverwort, *Marchantia inflexa* Nees & Mont., high light intensity produces relatively thicker thalli (analogous to true leaves, Groen *et al.* 2010a). In addition, pigmentation (Groen *et al.*, 2010b) and leaf/thallus traits (Groen *et al.* 2010a, in response to long-term light; Slate *et al.* 2017) differ between the sexes in two bryophyte species, potentially influencing SSS along light intensity gradients.

To test if light gradients can lead to SSS in bryophytes due to underlying functional sex differences, we used the thalloid liverwort, *M. inflexa*, as our model. While *M. inflexa* is considered a shade plant (Groen *et al*. 2010a,b), previous work showed that, though the sexes strongly overlap in occupied light conditions (intensity and canopy openness), the highest light conditions are occupied by males rather than females (Fuselier and McLetchie 2004). Because males produce sperm dorsally (more light exposed) and females produce eggs and sporophytes ventrally (less light exposed), selection for light protection might be higher in males than females during the sexual stage, which could result in a correlated response in the vegetative stage. Thus, we hypothesized that males would exhibit high light tolerance traits more than females. Because previous studies have also shown the sexes can be dimorphic prior to sexual reproduction (Groen *et al*. 2010b; Stieha *et al*. 2014), we thought it reasonable to test for sex differences among vegetative thalli, but we expected differences to be most pronounced among sexual thalli. This potential difference in high light tolerance could be achieved either by constitutive sexually dimorphic traits or sex-specific plastic responses to light intensity.

Further, vegetative thalli have a carbon acquisition function (photosynthesis), and this function more strongly aligns with female sexual thalli (offspring—i.e. sporophyte—support) than male sexual thalli (no offspring support). Thus, we reasoned that trait deviations between vegetative and sexual thalli would be greater for males than females with the exception of traits that facilitate unique female reproductive functions such as sperm capture. A caveat is that while bryophyte females are generally expected to have higher total sexual reproductive investment than males, males are expected to have higher pre-fertilization investment than females due to higher gamete production in males (McLetchie 1992; Stark *et al*. 2000).

Based on seed plant and other bryophyte patterns, we predicted that in response to high light and relative to females, males, especially in sexual thalli, would have lower total chlorophyll, higher chlorophyll *a*/*b* ratios (but see Groen *et al.* 2010b for a contrasting result), and higher carotenoid/chlorophyll ratios (i.e. pigmentation), and lower specific thallus area (STA; analogous to SLA) and greater dry matter content (i.e. carbon allocation). We also expected that males would differ more in these traits between vegetative and sexual thalli than females.

## Materials and methods

### Study species and stock population

**Figure 1.**
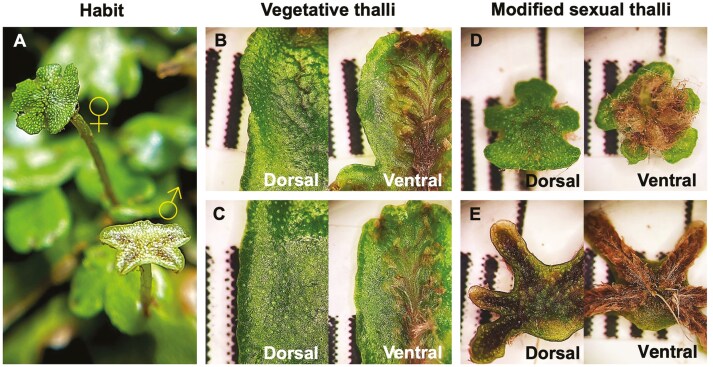
Thalli of *Marchantia inflexa*. (a) Intact thalli with female (top) and male (bottom) modified sexual thalli. Vegetative thalli are not in the focal plane. Dorsal and ventral surfaces of (b) a female vegetative thallus, (c) a male vegetative thallus, (d) a female sexual thallus, and (e) a male sexual thallus. Note the similarity between the dorsal surface of the modified sexual thallus and the dorsal surface of the vegetative photosynthetic surface and the loose scales in the centre of the ventral surface of the female modified sexual thallus. The scales in (b–e) are 1.0 mm.

*Marchantia inflexa* is a thalloid liverwort with distinct female and male individuals (Fig. 1a–e) and chromosomal sex determination (for *M. polymorpha*, Bischler 1986; Marks *et al.* 2019b). The species is distributed from southeast North America through the neotropics, typically occurring along streams. *Marchantia inflexa* grows as bifurcated, flattened vegetative thalli, approximately 5 mm wide, forming mats. On the island of Trinidad, where our stock population was collected, *M. inflexa* produces gametangia in modified thalli late in the dry season (March to May). The modified thallus of the sexually reproductive stage (analogous to seed plant sexual axes—modified stems/leaves) consists of a sex-organ-containing disk elevated on a stalk produced by the apical mericell of the vegetative thallus (archegoniophore and antheridiophore, female and male, respectively; Fig. 1a). In the present study, we use the term ‘sexual thallus’ to refer to the sex-organ-containing disk. The sexual thalli are sexually dimorphic. The female disk is dome-shaped with lobed edges. The dorsal surface has easily recognizable pores leading to air chambers with floors similar to those of flat vegetative thalli. Archegonia (with eggs) develop ventrally on the disk along with loosely attached scales (Fig. 1d). The male disk functions as a splash platform for sperm dispersal and is generally flat on top and star-shaped with several (four to six) finger-like projections. Antheridia (with sperm) are produced just beneath the dorsal surface of the disk. The floors of male disk air chambers are pointed due to the presence of antheridia. On the ventral side, scales are appressed (Fig. 1e). Typically, during the non-sexually reproductive season, thalli produce gemmae in gemma cups for asexual reproduction (Bischler 1984; for *M. polymorpha*, Schofield 1985). Males and females were collected in Trinidad from five streams: Quare River (10^o^40′29.7″N 61^o^11′47.5″), East (10^o^41′22.7″N 61^o^09′37.6″W) and West (10^o^40′41.6″N 61^o^10′03.0″W) branch of Turure River, North Oropuche River (10^o^40′09.4″N 61^o^08′14.9″W), and Rio Seco (10^o^43′29.3″N 61^o^02′01.3″W). These sites are environmentally variable and are known to have individuals that are genetically and phenotypically diverse (McLetchie and Puterbaugh 2000; Groen *et al.* 2010a; Brzyski *et al*. 2018; Marks *et al*. 2019a). Often, sub-populations of *M. inflexa* occur in distinct patches (occupied substrate surrounded by inhospitable substrate such as water). Plants were collected from distinct patches, and each isolate from one patch was considered a unique genotype (Brzyski *et al.* 2018). From these genotypes, a stock population was established approximately 7 years prior to this study and maintained in a glasshouse to reduce field effects. Each genotype was maintained in a separate ~59 mL pot. Because plants were watered by capillary action from below, isolating genotypes in separate pots prevented males and females from reproducing sexually. This would negate adaptation in the stock population. Voucher specimens are deposited at Missouri Botanical Garden (St. Louis, MO, USA, specimen numbers M0292113 and M092115) and at the National Herbarium of the Republic of Trinidad and Tobago (St. Augustine, Trinidad, specimen number TRIN34616).

### Experimental setup

To improve the generality of the results, we haphazardly chose 25 male and 25 female genotypes from the stock population with each sex represented by at least one genotype from each of the five origin streams. No two members of the same sex originated from the same patch in Trinidad. Two thallus tips (~1 cm in length) from each genotype were planted separately in ~59 mL pots with local (Lexington, KY, USA) steam-sterilized topsoil. One genotype clone was placed in each of two light intensity treatments for a total of 100 pots. Pots were fitted with neutral density acetate filters to produce high- and low-light treatments and were placed randomly in a 10 × 10 pot configuration on a capillary watering mat in a glasshouse to assure constantly moist soil. Though the manufacturer reports that the filters reduce all light wavelengths similarly, our own measurements (USB2000 Miniature Spectrometer, Ocean Optics Inc., Dunedin, FL, USA) indicated the filters absorbed relatively more from 400 to 560 nm in the visible spectrum, but this was consistent between filters and proportional to total light reduction. The high light filters (clear) transmitted about 85% of radiation, and the low light filters transmitted about 50% of solar radiation (catalogue numbers R00 and 209, respectively, www.leefiltersusa.com). Transmittance was estimated using a LI-1000 with a quantum sensor (Li-Cor, Lincoln, NE, USA). While day length is more variable in Lexington, KY, USA, than the field collection sites, the experimental glasshouse plants’ gross morphology and development appeared representative of field plants, and the glasshouse light levels overlap field light levels (Groen *et al*. 2010b).

We sampled plants after approximately 4 months of growth (October through January). Plants were sampled as they expressed sex over 11 weeks from 1 February to 18 April. We focused on two developmental stages: vegetative thalli (i.e. those neither connected to the stalk of a sexual thallus nor producing gemma cups) and sexual thalli above their supporting stalk (i.e. the antheridial or archegonial disk). Primarily, thalli with gemma cups would have been produced in the pre-sexual growth phase. Differences in sample timing could lead to different light environments experienced by the plants as day length changed, so we accounted for sampling time in our statistical analyses (see below).

### Pigment quantification

To estimate pigment concentration and ratios, we sampled vegetative and sexual thalli. These samples were placed directly into a well plate filled with distilled water, which ensured plants were fully hydrated prior to assessment. Before pigment extraction, samples were blotted with a paper towel and weighed to the nearest 0.1 mg. Pigments (chlorophylls and carotenoids) were extracted without maceration using 500 µl of methanol in individual centrifuge tubes (1 ml) at 60°C in a water bath for 1 hour. Extracted samples were then dried at 50°C for 24 hours and weighed to the nearest 0.0001 mg (Cahn microbalance) to obtain dry weight. Methanol is not an effective solvent for extracting carbohydrates, proteins, and very non-polar molecules (Christie 1993; Wong *et al*. 2004; Ganeva *et al*. 2015; Galvão *et al*. 2016; Wang *et al*. 2019). Thus, we expected other molecules dissolved in the methanol would have a minimal impact on dry mass estimates of extracted tissue. Pigment concentrations were estimated using absorbances from a well-plate reader spectrophotometer (GENios Plus, Tecan Trading AG, Switzerland). Because the well-plate reader only uses specific wavelengths for absorbances (we used 660 and 650 nm for chlorophylls and 465 nm for carotenoids), we developed equations through a pilot experiment to convert our pigment estimates to those of standard equations from Wellburn (1994) (see Supplementary Appendix and Supplementary Table A1) in a similar manner to Groen *et al.* (2010b) . Pigment content was expressed on a dry mass basis (total chlorophyll and total carotenoids) and as ratios (chlorophyll *a/b* ratio and total carotenoids/total chlorophyll).

### Thallus (vegetative or sexual) trait measurements

Using the same samples as for pigment estimation, we calculated thallus sample upper surface area after pigment extraction but before drying using a dissecting microscope with a camera and a Macintosh computer with the public domain NIH Image software (developed at the US National Institutes of Health and available on the web at https://imagej.net/nih-image/). Because they are nearly flat, we measured the area of vegetative thalli of both sexes and male sexual thalli directly from photographs taken through a dissecting microscope. We used the surface area of half a sphere as a reasonable and expedient surface area estimate of the female disk because its surface is dome-shaped but complex with marginal folds. Using our wet mass, dry mass, and upper surface area estimates, we calculated thallus dry matter content (TDMC) as dry mass/wet mass (analogous to leaf dry matter content) and STA as surface area/dry mass (analogous to SLA) for vegetative and sexual thalli.

### Statistical analyses

All statistical analyses were performed in SAS Studio 3.8 (SAS Institute, Cary, NC, USA).

We tested for overall effects of sex, light, and thallus type (vegetative or sexual) on the dependent variables considered together (chlorophyll, carotenoids, chlorophyll *a/b* ratio, carotenoid/chlorophyll ratio, TDMC, and STA) using a MANOVA in SAS’s GLM procedure. The model included sex, light intensity, and thallus type main effects along with all two-way interactions and the three-way interaction among these, and Type III sums of squares were used. Consistent with field observations (McLetchie and Puterbaugh 2000; Fuselier and McLetchie 2004), sexual thalli development was asynchronous, and plants were assessed on five different dates (1, 8, and 22 February; 28 March; and 18 April). Thus, we also included sampling date as a categorical covariate. Actual sample sizes for this and subsequent analyses were smaller than the total number of male and female genotypes (25 each) for reasons including plant death within a treatment (3 females in low light and 3 males in high light), failure to produce sexual thalli during the study period, and lack of suitable vegetative thalli for examination. Actual sample sizes were: in low light, females: 18 vegetative and 10 sexual thalli, and males: 22 vegetative and 20 sexual thalli; in high light, females: 21 vegetative and 13 sexual thalli, and males: 20 vegetative and 16 sexual thalli. Preliminary multivariate and univariate analyses suggested no significant effects of plant origin stream, so this potential block effect was excluded from all subsequent analyses. To test for correlations among dependent variables, we used partial correlations based on the MANOVA residuals. Normality assumptions were visually checked using histograms generated by SAS’s SGPLOT procedure, and STA, total chlorophyll, total carotenoids, and total carotenoids/total chlorophyll were log-transformed to improve normality for the MANOVA and subsequent univariate analyses.

For each dependent variable, we performed univariate analyses using generalized linear mixed models in SAS’s GLIMMIX procedure. We tested the effects of sex, light intensity, and thallus type along with all possible interactions among these on each dependent variable and again used Type III sums of squares. Sampling date was again included as a categorical covariate. Because a clone of each genotype was included in each light intensity, genotype and the interaction between genotype and light intensity were included as random effects. Where main effects and interactions were significant, we performed pairwise protected least significant difference (LSD) comparisons to examine the interaction pattern. Because we hypothesized that sex differences would be more pronounced in sexual thalli and because the MANOVA indicated a significant multivariate sex-by-thallus type interaction, we explored pairwise comparisons between thallus types within sex and between sexes within thallus type even when the univariate sex-by-thallus type interaction was not significant. We also calculated percent difference between sexual and vegetative thalli by subtracting the vegetative mean from the sexual mean within the sexes and dividing the result by the average of vegetative and sexual means. There was also a significant multivariate thallus type by light interaction, but we explored pairwise comparisons of light effects within thallus types only when univariate analyses also indicated a significant thallus type by light interaction.

## Results

### Multivariate analysis

The partial correlations showed that TDMC was significantly negatively correlated with STA, total chlorophyll per dry mass, and total carotenoids per dry mass. These three traits (STA, chlorophyll, and carotenoids) were significantly positively correlated with each other. Chlorophyll *a/b* ratio was significantly positively correlated with carotenoid/chlorophyll ratio. Finally, carotenoid/chlorophyll ratio was negatively correlated with chlorophyll (Table 1).

**Table 1.** Partial correlation coefficients among traits of *Marchantia inflexa* derived from the MANOVA residuals.

	Carotenoids	*a/b* ratio	car/chl	TDMC	STA
Chlorophyll	0.98^****^	0.0075	−0.30^***^	−0.63^****^	0.62^****^
Carotenoids		0.15	−0.083	−0.64^****^	0.65^****^
*a/b* ratio			0.61^****^	0.032	0.11
car/chl				0.097	0.029
TDMC					−0.74^****^

Dependent variables: chlorophyll (μg/mg), carotenoids (μg/mg), *a/b* ratio is chlorophyll *a* divided by chlorophyll *b*; car/chl is carotenoids divided by chlorophyll. STA, specific thallus area or area per dry mass; TDMC, thallus dry matter content.

^****^ *P* < 0.0001,

^***^ *P* < 0.001.

In the multivariate analysis, sex, light, and thallus type significantly affected the dependent variables (total chlorophyll per dry mass, total carotenoids per dry mass, chlorophyll *a/b* ratio, total carotenoids/total chlorophyll, TDMC, and STA). The two-way interactions between sex and thallus type (vegetative or sexual) and between thallus type and light were also significant, but the interactions between sex and light and the three-way interaction of sex, light, and thallus type were not significant (Table 2). The latter two interactions were also not significant in the subsequent univariate analyses (not shown). Thus, though included in our models, the interactions between sex and light and among sex, light, and thallus type will not be discussed further.

**Table 2.** MANOVA results.

Source	Wilks’ λ	*F*	*df*	*P*
Sex	0.68	11.24	5, 119	<0.0001
Light	0.60	15.92	5, 119	<0.0001
Thallus type	0.44	30.21	5, 119	<0.0001
Sex × light	0.98	0.52	5, 119	0.76
Sex × thallus type	0.68	11.04	5, 119	<0.0001
Light × thallus type	0.87	3.61	5, 119	0.0045
Sex × light × thallus type	0.98	0.53	5, 119	0.75

The analysis considered effects and interactions of sex (male or female), light (high or low), and thallus type (vegetative or sexual) on all dependent variables (chlorophyll, carotenoids, chlorophyll *a*/*b* ratio, carotenoid/chlorophyll ratio, thallus dry matter content, and specific thallus area) in *Marchantia inflexa*.

We explored the interaction of sex and thallus type using multivariate contrasts, which indicated that female and male sexual thalli differed from one another (Wilks’ λ = 0.57, *F*_*5*, 119_ = 18.22, *P* < 0.0001), while female and male vegetative thalli did not (Wilks’ λ = 0.98, *F*_*5*, 119_ = 0.55, *P* = 0.74). Furthermore, while the vegetative and sexual thalli were different within both females (Wilks’ λ = 0.82, *F*_*5*, 119_ = 5.07, *P* = 0.0003) and males (Wilks’ λ = 0.35, *F*_*5*, 119_* *= 44.62, *P *< 0.0001), the difference was greater for males than for females (based on the *F* values).

We also examined the light-by-thallus type interaction using multivariate contrasts, and found that for thallus type, low and high light were more different from each other for vegetative thalli (Wilks’ λ = 0.57, *F*_*5*, 119_ = 18.00, *P* < 0.0001) compared to sexual thalli (Wilks’ λ = 0.87, *F*_*5*, 119_ = 3.56, *P* = 0.0049). In addition, within light levels, vegetative and sexual thalli were more different from each other (based on the *F* values) in low light (Wilks’ λ = 0.51, *F*_*5*, 119_ = 22.61, *P* < 0.0001) compared to high light (Wilks’ λ = 0.65, *F*_*5*, 119_* *= 12.69, *P *< 0.0001).

### Univariate analyses

#### Total chlorophyll and carotenoids per dry mass

**Figure 2.**
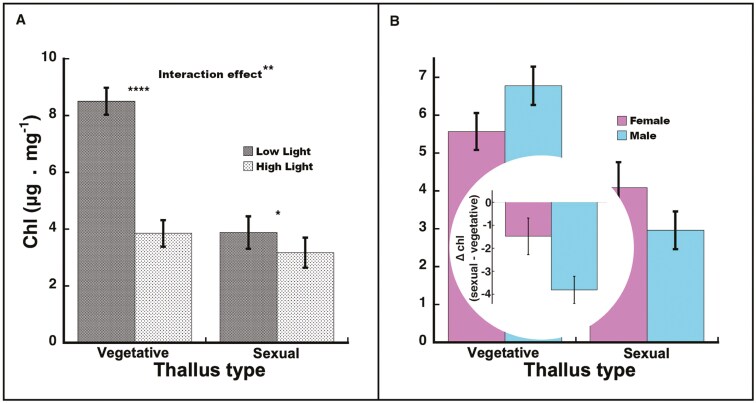
Chlorophyll per dry weight in *Marchantia inflexa*. (a) By light and thallus type (vegetative or sexual). There was a significant interaction between light and thallus type. While both thalli types decreased chlorophyll from low to high light, vegetative thalli decreased more than sexual. (b) By sex and thallus type. Although the interaction was not significant, the mean difference (sexual—vegetative) pattern was consistent with males having a greater change than females. Figure inset: Difference between sexual and vegetative thalli in chlorophyll per dry weight. Values are least square mean ± SE. ****P < 0.0001, **P < 0.01, *P < 0.05.

The univariate results for chlorophyll and carotenoids per dry mass mirrored one another and carotenoid figures are not shown (but see Supplementary Fig. S1). Overall, plants in high light had significantly lower chlorophyll and carotenoids than those in low light (chlorophyll: *F*_1, 40_ = 44.54, *P *< 0.0001; carotenoids: *F*_1, 40_* *= 61.27, *P *< 0.0001). Overall, vegetative thalli had significantly higher chlorophyll and carotenoids than sexual thalli (chlorophyll: *F*_1, 35_* *= 52.97, *P *< 0.0001; carotenoids: *F*_1, 35_* *= 56.82, *P *< 0.0001). A significant light-by-thallus type interaction (chlorophyll: *F*_1, 35_* *= 12.21, *P *= 0.0013; Fig. 2a; carotenoids: *F*_1, 35_* *= 9.59, *P *= 0.0038) demonstrated that the light effect was greater for vegetative thalli (*t*_35_ ≥ 7.93, *P *< 0.0001 for both) than for sexual thalli (chlorophyll: *t*_35_ = 2.24, *P *= 0.032; carotenoids: *t*_35_ = 3.01, *P *= 0.0048). The overall sex effect on both pigments (chlorophyll: *F*_1, 35_* *= 0.00, *P *= 0.99; carotenoids: *F*_1, 35_* *= 0.19, *P *= 0.67) and the sex effect within each thallus type (|*t*_35_| ≤ 1.23, *P *> 0.22 for all) were not significant. The sex-by-thallus type interactions were not significant (chlorophyll: *F*_1, 35_* *= 2.18, *P *= 0.15; carotenoids: *F*_1, 35_* *= 1.62, *P *= 0.21). The difference between vegetative and sexual thalli for both pigments was significant within both females and males (for all comparisons, *t*_35_ ≤ −3.76, *P *≤ 0.0006; Fig. 2b; Supplementary Fig. S1b) with the average percent difference being larger for males in both cases (chlorophyll: ♂ −78.4% vs ♀ −30.6%; Fig. 2b inset; carotenoids: ♂ −75.4% vs ♀ −37.6%; Supplementary Fig. S1b inset), which should be interpreted cautiously because the sex-by-thallus type interactions were not significant.

#### Chlorophyll *a/b* ratio

**Figure 3.**
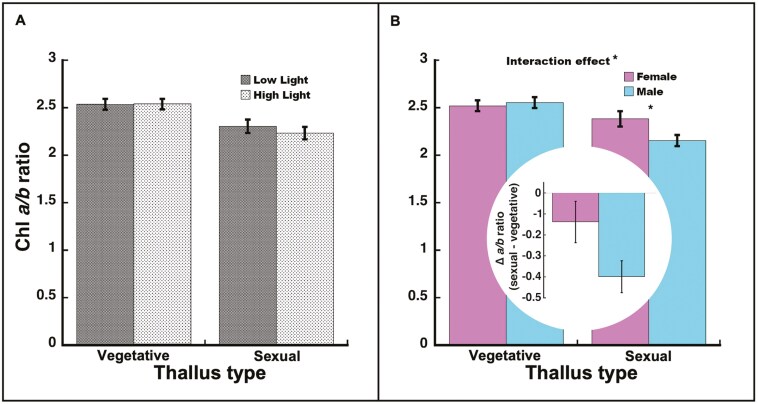
Chlorophyll *a/b* ratio in *Marchantia inflexa*. (a) By light and thallus type (vegetative or sexual). In both vegetative and sexual thalli, chlorophyll *a/b* ratio did not differ by light. (b) By sex and thallus type. There was a significant interaction between sex and thallus type. Males decreased (sexual—vegetative thallus) more than females. Figure inset: Difference between sexual and vegetative thalli in chlorophyll *a/b* ratio. Values are least square mean ± SE. *P < 0.05.

Chlorophyll *a/b* ratio was not significantly affected by light (*F*_1, 41_* *= 0.32, *P *= 0.58). Vegetative thalli had significantly higher chlorophyll *a/b* ratios than sexual thalli (*F*_1, 35_* *= 17.91, *P *= 0.0002). The light-by-thallus type interaction was not significant (*F*_1, 35_* *= 0.41, *P *= 0.53; Fig. 3a). The sex effect was not significant (*F*_1, 35_* *= 2.27, *P *= 0.14), but the sex-by-thallus type interaction was significant (*F*_1, 35_* *= 4.55, *P *= 0.040; Fig 3b), with female sexual thalli having higher chlorophyll *a/b* ratios than male sexual thalli (*t*_35_ = 2.33, *P *= 0.026). The sex effect was not significant for vegetative thalli (*t*_35_ = −0.41, *P *= 0.69). Males exhibited a significantly higher chlorophyll *a/b* ratio for vegetative thalli than for sexual thalli (*t*_35_ = −5.23, *P *< 0.0001), but the female thallus types did not differ (*t*_35_ = −1.40, *P *= 0.17), corresponding to a higher percent difference between vegetative and sexual thalli for males vs females (♂ −17.0% vs ♀ −5.6%; Fig. 3b inset).

#### Carotenoid/chlorophyll ratio

**Figure 4.**
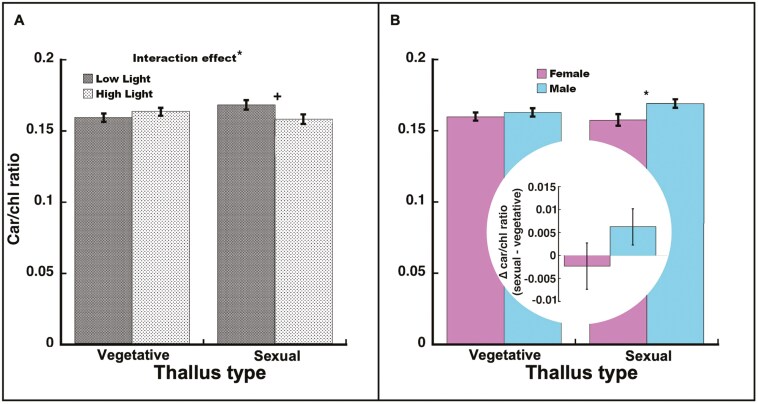
Carotenoid/chlorophyll ratio in *Marchantia inflexa*. (a) By light and thallus type (vegetative or sexual). There was a significant interaction between light and thallus type. In vegetative thalli, carotenoid/chlorophyll ratio did not differ by light, while in sexual thalli, carotenoid/chlorophyll ratio was marginally higher in low light than high light. (b) By sex and thallus type. Although the interaction was not significant, the mean difference (sexual—vegetative) pattern was consistent with males having a greater change than females. In vegetative thalli, the sexes did not differ, while in sexual thalli, males had greater carotenoid/chlorophyll ratio than females. Figure inset: Difference between sexual and vegetative thalli in carotenoid/chlorophyll ratio. Values are least square mean ± SE. *P < 0.05, ^+^*P *= 0.0509.

The light and thallus type effects on carotenoid/chlorophyll ratio were not significant (*F*_1, 41_* *= 0.72, *P *= 0.40 and *F*_1, 35_* *= 0.18, *P *= 0.67, respectively), but their interaction was significant (*F*_1, 35_* *= 5.25, *P *= 0.028; Fig. 4a), with sexual thalli in high light having marginally lower carotenoids/chlorophyll ratios than sexual thalli in low light (*t*_35_ = −2.02, *P *= 0.0509). There was no significant difference in the carotenoid/chlorophyll ratio between high and low light vegetative thalli (*t*_35_ = 1.03, *P *= 0.31), but this non-significant pattern was opposite in relation to light compared to sexual thalli. Overall, males had significantly higher carotenoid/chlorophyll ratios than females (*F*_1, 35_* *= 5.10, *P *= 0.030), but within thallus type, there was no sex effect in vegetative thalli (*t*_35_ = −0.68, *P *= 0.50) while males had higher carotenoid/chlorophyll ratios than females in sexual thalli (*t*_35_ = −2.40, *P *= 0.022). However, the means for the sexes were in the same direction for vegetative thalli as for sexual thalli. The sex-by-thallus type interaction was not significant (*F*_1, 35_* *= 2.15, *P *= 0.15; Fig. 4b). When comparing thallus types within either males (*t*_35_ = 1.52, *P *= 0.14) or females (*t*_35_ = −0.64, *P *= 0.53), there was no significant difference, but the percent difference was larger for males (♂ 3.7% vs ♀ −1.4%; Fig. 4b inset).

#### Thallus dry matter content

**Figure 5.**
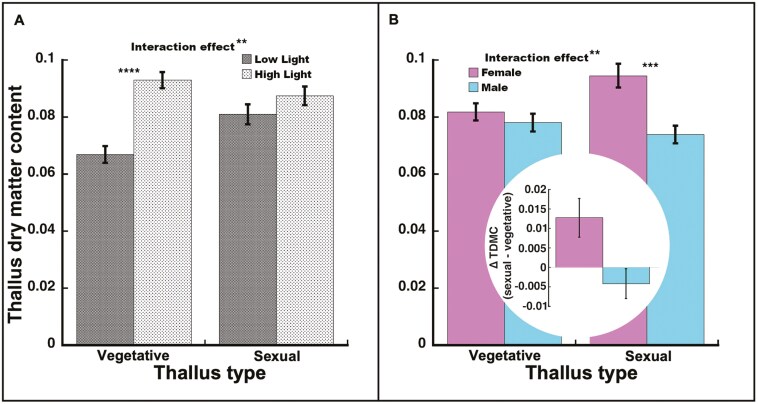
Thallus dry matter content (TDMC—dry weight/fresh weight) in *Marchantia inflexa*. (a) By light and thallus type (vegetative or sexual). There was a significant interaction between light and thallus type. In vegetative thalli, TDMC was lower in low light than high light, while in sexual thalli, TDMC did not differ by light. (b) By sex and thallus type. There was a significant interaction between sex and thallus type. Females increased (sexual—vegetative thallus) while males non-significantly decreased. Figure inset: Difference between sexual and vegetative thalli in TDMC. Values are least square mean ± SE. ****P < 0.0001, ***P < 0.001, **P < 0.01.

Overall, plants in high light had significantly higher TDMC than those in low light (*F*_1, 40_* *= 32.68, *P *< 0.0001). Thallus type did not significantly affect TDMC (*F*_1, 35_* *= 1.79, *P *= 0.19), but there was a significant light-by-thallus type interaction (*F*_1, 35_* *= 11.93, *P *= 0.0015; Fig. 5a). Vegetative thalli had a higher TDMC in high light vs low light (*t*_35_ = 7.10, *P *< 0.0001). For sexual thalli, the difference between light intensities was not significant (*t*_35_ = 1.50, *P *= 0.14) but similar in direction. Overall, females had a higher TDMC than males (*F*_1, 35_* *= 11.50, *P *= 0.0017). The sex-by-thallus type interaction (*F*_1, 35_* *= 7.56, *P *= 0.0094; Fig 5b) was significant. Female sexual thalli had higher TDMC than male sexual thalli (*t*_35_ = 4.01, *P *= 0.0003), but there was no significant difference between female and male vegetative thalli (*t*_35_ = 0.88, *P *= 0.38). When comparing thallus type within the sexes, female sexual thalli had higher TDMC than vegetative thalli (♀ 14.4% difference, *t*_35_ = 2.56, *P *= 0.015), while male sexual thalli did not differ significantly from their vegetative thalli (♂ −5.5% difference, *t*_35_ = −1.09, *P *= 0.29; Fig 5b inset).

#### Specific thallus area

**Figure 6.**
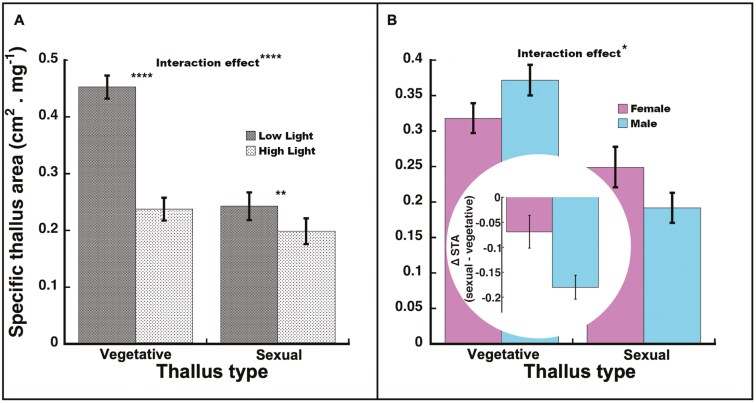
Specific thallus area (STA, area/dry weight) in *Marchantia inflexa*. (a) By light and thallus type (vegetative or sexual). There was a significant interaction between light and thallus type. In vegetative thalli, STA was higher in low light than in high light, while in sexual thalli, STA did not differ by light. (b) By sex and thallus type. There was a significant interaction between sex and thallus type. Males decreased (sexual—vegetative thallus) more than females. Figure inset: Difference between sexual and vegetative thalli in STA. Values are least square mean ± SE. ****P < 0.0001, **P < 0.01, *P < 0.05.

Overall, plants in high light had lower STA (*F*_1, 40_* *= 96.79, *P *< 0.0001), and sexual thalli had lower STA than vegetative thalli (*F*_1, 35_* *= 71.94, *P *< 0.0001). The light-by-thallus type interaction was significant (*F*_1, 35_* *= 18.46, *P *= 0.0001; Fig. 6a). Though significant and in the same direction in both vegetative and sexual thalli, the difference between low and high light was greater in vegetative thalli (*t*_35_ = −10.91, *P *< 0.0001) than sexual thalli (*t*_35_ = −3.52, *P *= 0.0012). Overall, there was no effect of sex on STA (*F*_1, 35_* *= 0.040, *P* = 0.84), but the sex-by-thallus type interaction was significant (*F*_1, 35_* *= 7.05, *P *= 0.012; Fig. 6b). The sex difference for STA was not significant for sexual thalli (*t*_35_ = 1.64, *P *= 0.11) or vegetative thalli (*t*_35_ = −1.60, *P *= 0.12), but males had a greater difference (♂ −63.9%, *t*_35_ = −9.32, *P *< 0.0001) between their vegetative and sexual thalli than females (♀ −24.3%, *t*_35_ = −3.81, *P *= 0.0005; Fig. 6b inset).

## Discussion

We expected sexual thalli to be more sexually dimorphic than vegetative thalli, and our multivariate analysis supported this expectation. In addition, the multivariate difference between vegetative and sexual thalli was greater for males than females, and individual trait analyses were also consistent with this except for one trait (TDMC) where females differed more. Examining these sex-specific trait changes between thallus types provides additional insight into the importance of these traits for sex function, which we explore below. While not sexually dimorphic, vegetative thalli across both sexes exhibited overall greater plasticity to light than sexual thalli, suggesting constraints from specialization for sexual reproduction. As expected, for most traits, vegetative thalli largely responded to increasing light like vascular plant leaves. However, chlorophyll *a/b* and carotenoid/chlorophyll ratios did not increase with light as expected. Despite sexually dimorphic patterns, sex-specific light responses were absent for all traits, which does not support our prediction of greater sex differences at high light intensities or explain previous field sex-specific canopy openness/light distributions (Fuselier and McLetchie 2004).

### Integrating sex differences in developmental changes with sex function

To infer links between sexual dimorphism and sex function, we explored sexual dimorphisms in two ways, more traditionally by comparing traits directly and alternatively by comparing sex differences in trait changes between developmental stages (i.e. two thallus types). For *M. inflexa* males and females, vegetative thallus functions (growth and survival) overlap more strongly than sexual thallus functions (males: sperm production/dispersal vs females: egg production, sperm capture, and sporophyte support). Thus, sexual thalli should manifest higher levels of sexual dimorphisms. Consistent with these ideas, we found that vegetative thalli were not sexually dimorphic for any trait, but male and female sexual thalli differed in three of six traits (chlorophyll *a/b* and carotenoid/chlorophyll ratios and TDMC). Additionally, because female sexual thalli invest more overall in sexual reproduction over a longer period (growth and survival) than male sexual thalli, female stages should have more functional overlap and trait similarity than male stages. We found greater overlap for females in chlorophyll *a/b* ratio and STA with some hints of greater overlap in chlorophyll, carotenoids, and carotenoid/chlorophyll ratio relative to males. In contrast, TDMC differed more between thallus types for females than males. In our following discussion, we use both comparison types for deeper insight into the link between traits and sex-specific functions while generating additional testable predictions.

The traditional comparison between female and male sexual thalli suggests the higher TDMC of females is due to dense, exaggerated ventral scales (unique to female sexual thalli; Fig. 1d), and this pattern was reinforced by the alternative approach. The larger TDMC change between stages in females than males firmly links TDMC to female sex function. These scales are most likely acting as a structure for capturing sperm-laden water as previously suggested for *M. polymorpha* (Pressel and Duckett 2019). Analogous sperm or pollen capturing female structures were proposed for mosses (Moore *et al*. 2016) and seed plants (Niklas 1985a,b; Niklas and Buchmann 1985; Bond and Maze 1999), respectively.

Unlike females, sexual thalli in males are self-shaded by a reddish, anthocyanin-like pigment (probably riccionidin A, an auronidin; Fig. 1d–e), which could protect dorsally developing sperm from damaging light (Landi *et al*. 2015; Albert *et al*. 2018; Berland *et al*. 2019). Furthermore, the dorsal photosynthetic layer is disrupted in male sexual thalli by antheridia, whereas this layer in female sexual thalli is not disrupted (Schofield 1985). This might explain both the greater difference between male stages in chlorophyll *a/b* ratio relative to females and the greater carotenoid/chlorophyll ratio of male vs female sexual thalli. The former is due to a greater reduction of chlorophyll *a/b* ratios in male sexual thalli (compared to male vegetative thalli) relative to the reduction in females. Reduced chlorophyll *a/b* ratios are associated with lower light conditions (Givnish 1988), and although not observed in the present study for vegetative thalli, a previous study on *M. inflexa* found the expected low light effect (Groen *et al.* 2010b). Furthermore, vascular plant leaves self-shaded by anthocyanins show reduced chlorophyll *a/b* ratios along with higher dark respiration or reduced photosynthesis (Hughes *et al*. 2014; Yu *et al*. 2019). If elevated carotenoid/chlorophyll ratios are due to carotenoid accessory function (discussed further below), this could also link to self-shading. To test if higher riccionidin A leads to lower chlorophyll *a/b* ratios and increased carotenoid/chlorophyll ratios, quantification of riccionidin A is needed.

Though not sexually dimorphic in the traditional sense, STA showed a sexually dimorphic pattern with males differing more between stages than females. There was also a lack of the expected negative relationship between TDMC and STA (Wilson *et al*. 1999; Table 1) across the male stages (Figs 5b and 6b). These patterns suggest a link between STA and male sex function that requires further study. The linkage might be due to resource demands of a male sexual thallus (sperm production and sturdy splash platform construction for sperm dispersal) and its potentially impaired photosynthetic capacity through self-shading and a disrupted photosynthetic layer to meet those demands. Thus, we expect higher and more costly pre-fertilization investment for males than females as suggested in other bryophytes, despite higher overall female reproductive investment (McLetchie 1992; Stark *et al.* 2000; Horsley *et al.* 2011). While low SLA (the vascular plant analogue of STA) generally indicates less photosynthetic area is needed to support leaf biomass (Poorter *et al*. 2019), high carbohydrate translocation could produce a sink with low SLA similar to a structure with high photosynthetic capacity. Analogous SLA shifts between sources and sinks occur in vascular plants (Poorter *et al*. 2009). Thus, we posit that, for male sexual thalli, higher upfront sexual reproductive investment and compromised photosynthetic capacity result in more reliance on translocated carbohydrates via photosynthate conducting cells (Ligrone and Duckett 1994; Ligrone *et al*. 2000; Edwards *et al.* 2003), leading to low STA relative to other thallus types pre-fertilisation. Testing these ideas requires quantifying non-structural carbohydrates and photosynthesis in sexual and adjacent thalli of both sexes before and after fertilization or sperm dehiscence. Before fertilization and compared to female thalli, we expect reduced non-structural carbohydrates in male thalli attached to sexual thalli, but this could be reversed after fertilization and sperm dehiscence.

### Vegetative thalli respond to light like vascular plant leaves

Overall, vegetative thalli light responses, with a few noteworthy exceptions, reflected plasticity similar to vascular plant leaves and other bryophytes. As expected, low light thalli had more chlorophyll and carotenoids per dry mass. Our chlorophyll results are consistent with previous work on *M. inflexa* (Groen *et al.* 2010b). In low light, plants are light-limited and generally invest more in light-gathering pigments (Marschall and Proctor 2004; Niinemets 2010; Tobias and Niinemets 2010). In high light where excessively excited pigments damage membranes (Ort 2001; Fan *et al.* 2020), plants are more carboxylation-limited (Huntingford and Oliver 2021). Higher TDMC and lower STA in high light were also expected. Higher dry matter content can relate to higher photosynthesis (Min *et al.* 2021), and brighter light penetrates deeper into the leaf/thallus, permitting a thicker mesophyll/photosynthetic layer (Groen *et al.* 2010b; Poorter *et al.*, 2019). However, chlorophyll *a/b* and carotenoid/chlorophyll ratios did not respond as expected (i.e. to increase with light), remaining puzzlingly non-plastic to light, which contrasts previous work on *M. inflexa* (Groen *et al.* 2010b), other bryophytes (Marschall and Proctor 2004), and vascular plants (Sarijeva *et al.* 2007; Poorter *et al.* 2019).

### Plasticity to light in vegetative and sexual thalli

When considering all traits, sexual thalli were less plastic to light than vegetative thalli. For chlorophyll, carotenoids, TDMC, and STA, light responses in sexual thalli were significant and directionally similar to vegetative thalli but of smaller magnitudes (i.e. reduced plasticity). For sexual thalli, dampened plasticity may be a cost of specialization for sexual reproduction and thus relate to structural constraints (a tradeoff). For vegetative thalli, altering non-photosynthetic and photosynthetic tissue to optimize carbon acquisition might be more easily done compared to sexual thalli.

Interestingly, for one trait, carotenoid/chlorophyll ratio, sexual thalli were more plastic to light than vegetative thalli. Sexual thalli reduced the ratio while vegetative thalli did not significantly respond to increasing light. This pattern contrasts the apparent photoprotective increase in carotenoid/chlorophyll ratio with increasing light in several vascular plants and bryophytes (Hamerlynck *et al.* 2002; Dunn and Robinson 2006; Tobias and Niinemets 2010; Glime 2017; Fan *et al.* 2019). In our study, the negative partial correlation between chlorophyll and carotenoid/chlorophyll ratio (i.e. as chlorophyll decreases, carotenoids relatively increase) across sexes and thallus types suggests a similar photoprotective role for carotenoids. However, if carotenoids were photoprotective as expected, then the ratio should have increased for both thallus types as light increased. The pattern from sexual thalli is more consistent with an accessory function (well known for carotenoids; Bartley and Scolnik 1995), and light levels were possibly not high enough to warrant increased photoprotective carotenoid production. Here, total carotenoids include xanthophyll cycle pigments and others such as beta-carotene. Quantifying different carotenoid forms should clarify whether *M. inflexa* is adjusting carotenoid/chlorophyll ratios for accessory or photoprotective functions.

### Sexually dimorphic traits not clearly linkable to male higher light tolerance

Our study was based on previous field work in Southeastern and South-central USA and the island of Trinidad, showing that males occupy a wider range of light or canopy openness environments, including higher light/openness, than females (i.e. SSS; Fuselier and McLetchie 2004). Those results suggested males should either be more plastic to light or have traits more consistent with higher light adaptation than females. Given field variability, we anticipated clearer sex-specific light responses in a controlled glasshouse experiment, but despite finding sexually dimorphic traits, our results were not consistent with our expectations and do not explain the apparent field SSS. Furthermore, even though males change more between developmental stages than females, these differences were not light-related nor do they clearly give males an advantage in higher light. If male sexual thalli are more of a carbon sink than those of females prior to fertilization, one might expect males to survive better in high light, but that would not explain why females should not also occupy those same habitats. One possible explanation is that traits are dimorphic in one region (USA) and not the other (Trinidad). Population variation in dimorphism has been previously reported for *M. inflexa* (Brzyski *et al.* 2014; . Marks *et al.* 2019a). In the present study, no US populations, which might exhibit sex-specific light responses, were included. Other possibilities to explain SSS in *M. inflexa* include sex-specific responses to other light-correlated environmental factors (temperature, moisture, and intersexual competition). Although a previous study showed females had higher dehydration tolerance than males (Marks *et al*. 2019a), there was no moisture-related SSS. One rarely discussed alternative explanation is that SSS can result from combinations of random colonization events, clonal growth, and disturbances or local extinctions. Mathematical modelling of *M. inflexa* population sex ratios using sex-specific life histories and only disturbance/extinction gradients results in SSS at local population and metapopulation scales (McLetchie *et al*. 2002; Crowley *et al*. 2005; Garcia-Ramos *et al*. 2007; Stieha *et al*. 2017). If disturbance correlates positively with light (higher heat and desiccation events), then because males are predicted to have higher asexual colonization ability (Stieha *et al*. 2014), this could explain why males tend to occupy higher light habitats than females.

## Conclusion

Comparing sex differences in trait changes between developmental stages, considering multiple traits simultaneously, provided additional insight into how these traits link to sex function compared to traditional trait comparisons alone. Here, we found that while the sexes do not respond differently to light, males generally need to modify pigment and biomass allocation traits more compared to females to move from the vegetative to the sexual stage. Taken together, several sexually dimorphic traits and sex-specific changes between stages suggest that male sexual thalli may require more support than their female counterparts before sperm dehiscence and female investment in offspring (sporophytes). Future work should target the functional significance of sexually dimorphic patterns identified here by linking these to other traits such as photosynthesis or carbohydrate translocation and how these might vary before and after fertilization.

## Supplementary Material

plaf010_suppl_Supplementary_Materials

## Data Availability

The data for this study are freely available at the following link: https://figshare.com/s/9e9971cbc62508bfa2e1
