# Supplementary material for: Males differ more between developmental stages than females, and plasticity to light is stage-dependent in the tropical plant Marchantia inflexa
Source: AoB Plants. 2025 Feb 22;17(2):plaf010. doi: 10.1093/aobpla/plaf010 (PMC11966608; doi:10.1093/aobpla/plaf010)
Supplement: plaf010_suppl_Supplementary_Materials [file plaf010_suppl_supplementary_materials.pdf]

Supporting Information

APPENDIX

We performed similar pilot experiments as Groen et al., (2010 *b*) to correlate estimates of chlorophylls *a* + *b* and carotenoids from a well-plate reader spectrophotometer (GENios Plus, Tecan Trading AG, Switzerland) with those of a standard spectrophotometer (Spectronic 601, Milton Roy Co., Rochester, NY, USA). Using a microplate reader dramatically reduces time needed to assess pigment extract absorbances, but the GENios Plus has a limited number of preset wavelengths to measure absorbance, which do not exactly match those of standard equations (Wellburn 1994). To correlate the GENios Plus with the standard spectrophotometer, we extracted pigments extracted from ~1.6 g (FW) of *Marchantia inflexa* thallus tissue sourced haphazardly from the stock population with 15 mL of methanol at 60°C for 1 h. We chose the temperature based on Devesa *et al*. (2007) and results of early pilot trials where thalli were translucent after incubation for 1 h. This extract was then diluted to concentrations of 5, 10, 15, 22, 25, 27, 30, 40, 50, 60, 70, 80, 90, and 100%. Absorbances of these solutions were taken at both wavelengths for equations for high resolution spectrophotometers (665.2, 652.4, and 470 nm) from Wellburn (1994) and at the nearest ones available from the GENios plus (660, 650, and 465 nm). We estimated chlorophylls *a* + *b* and total carotenoid concentration using the equations from Wellburn (1994) with absorbances from the spectrophotometer or the GENios plus. We used linear regression in Microsoft Excel (Microsoft Office Professional Plus 2019) to create an equation for each pigment to convert estimates using the GENios machine (independent variable) to estimates from the spectrophotometer (dependent variable; Table A1).

Table A1. Regression equations based on pigment concentration estimates (μg pigment · mL^{-1} solution) from ~1.6 g (FW) of *Marchantia inflexa* tissue using absorbances from a microplate reader ($E_{microplate}$) and a spectrophotometer (E_{spec}). All regressions were significant ($p < 0.0001$).

Pigment	Equation	R^2
Chlorophyll *a*	$E_{spec} = 2.14576 \times E_{microplate} + 0.05939$	0.999
Chlorophyll *b*	$E_{spec} = 3.71030 \times E_{microplate} - 0.02551$	0.998
Total Carotenoids	$E_{spec} = 1.29183 \times E_{microplate} + 0.01246$	0.997

Additional References

Devesa R, Moldes A, Diaz-Fierros F et al Extraction study of algal pigments in river bed sediments by applying factorial designs. Talanta 2007;72:1546–51. https://doi.org/10.1016/j.talanta.2007.02.006A

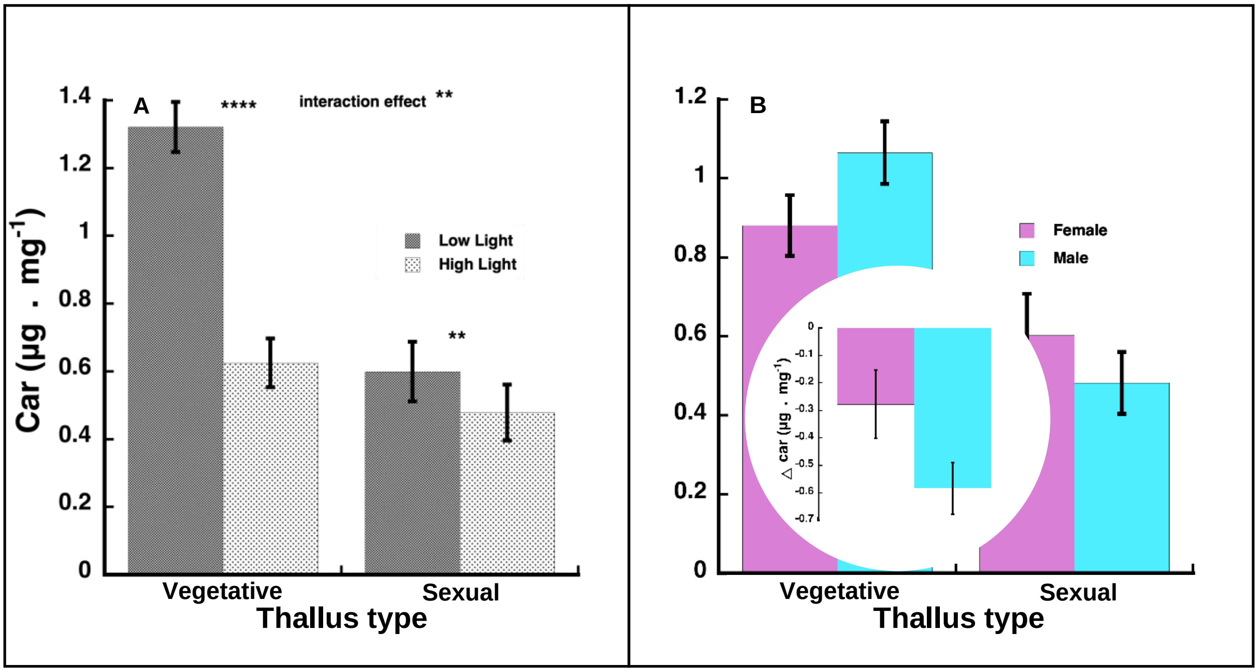

Figure S1. *Carotenoids* per *dry weight in* Marchantia inflexa. (A) by light and thallus type (vegetative or sexual). There was a significant interaction between light and thallus type. While both thalli types decreased carotenoids from low to high light, vegetative thalli decreased more than sexual. (B) by sex and thallus type. Although the interaction was not significant, the mean difference (sexual - vegetative) pattern was consistent with males having a greater change than females. Figure inset: Difference between sexual and vegetative thalli in carotenoids per dry weight. Values are least square mean ± SE. **** $p < 0.0001$, ** $p < 0.01$.
